# Comparing six cardiovascular risk prediction models in Haiti: implications for identifying high-risk individuals for primary prevention

**DOI:** 10.1186/s12889-022-12963-x

**Published:** 2022-03-19

**Authors:** Lily D. Yan, Jean Lookens Pierre, Vanessa Rouzier, Michel Théard, Alexandra Apollon, Stephano St Preux, Justin R. Kingery, Kenneth A. Jamerson, Marie Deschamps, Jean W. Pape, Monika M. Safford, Margaret L. McNairy

**Affiliations:** 1 Division of General Internal Medicine, Department of Medicine, Weill Cornell Medicine, New York, NY USA; 2 Center for Global Health, Weill Cornell Medicine, New York, NY USA; 3 Haitian Group for the Study of Kaposi’s Sarcoma and Opportunistic Infections (GHESKIO), Port-au-Prince, Haiti; 4 Collège Haïtien de Cardiologie, Port-au-Prince, Haiti; 5 Division of Cardiovascular Disease, Department of Medicine, University of Michigan, Ann Arbor, MI USA

**Keywords:** Cardiovascular diseases, Primary prevention, Cardiovascular Risk, Global health, Hypertension

## Abstract

**Background:**

Cardiovascular diseases (CVD) are rapidly increasing in low-middle income countries (LMICs). Accurate risk assessment is essential to reduce premature CVD by targeting primary prevention and risk factor treatment among high-risk groups. Available CVD risk prediction models are built on predominantly Caucasian risk profiles from high-income country populations, and have not been evaluated in LMIC populations. We aimed to compare six existing models for predicted 10-year risk of CVD and identify high-risk groups for targeted prevention and treatment in Haiti.

**Methods:**

We used cross-sectional data within the Haiti CVD Cohort Study, including 1345 adults ≥ 40 years without known history of CVD and with complete data. Six CVD risk prediction models were compared: pooled cohort equations (PCE), adjusted PCE with updated cohorts, Framingham CVD Lipids, Framingham CVD Body Mass Index (BMI), WHO Lipids, and WHO BMI. Risk factors were measured during clinical exams. Primary outcome was continuous and categorical predicted 10-year CVD risk. Secondary outcome was statin eligibility.

**Results:**

Sixty percent were female, 66.8% lived on a daily income of ≤ 1 USD, 52.9% had hypertension, 14.9% had hypercholesterolemia, 7.8% had diabetes mellitus, 4.0% were current smokers, and 2.5% had HIV. Predicted 10-year CVD risk ranged from 3.6% in adjusted PCE (IQR 1.7–8.2) to 9.6% in Framingham-BMI (IQR 4.9–18.0), and Spearman rank correlation coefficients ranged from 0.86 to 0.98. The percent of the cohort categorized as high risk using model specific thresholds ranged from 1.8% using the WHO-BMI model to 41.4% in the PCE model (χ^2^ = 1416, *p* value < 0.001). Statin eligibility also varied widely.

**Conclusions:**

In the Haiti CVD Cohort, there was substantial variation in the proportion identified as high-risk and statin eligible using existing models, leading to very different treatment recommendations and public health implications depending on which prediction model is chosen. There is a need to design and validate CVD risk prediction tools for low-middle income countries that include locally relevant risk factors.

**Trial registration:**

clinicaltrials.gov NCT03892265.

**Supplementary Information:**

The online version contains supplementary material available at 10.1186/s12889-022-12963-x.

## Background

Cardiovascular diseases (CVD) are rapidly increasing in low-middle income countries (LMICs), with ischemic heart disease, stroke, and peripheral artery disease amounting to over 17 million deaths in 2017 [1]. Furthermore, mortality and disability due to CVD have increased by 21.1% and 16.4%, respectively, over the past ten years [1]. Multiple risk factors contribute to the increase in CVD, including modifiable risk factors like high systolic blood pressure (SBP), hyperlipidemia, tobacco and alcohol use, poor diet, and physical inactivity [2]. In addition, many LMICs may have additional poverty-related CVD risk factors like heavy metal and severe air pollution, food insecurity, and increased allostatic load from stress, social isolation, and political strife [3, 4].

Accurate CVD risk prediction to target use of statins and antihypertensives in primary CVD prevention is essential to reduce premature disease, especially in a LMIC country like Haiti where CVD leads mortality at 26.5% of all adults deaths, and where there are significant resource-constraints [5, 6]. Currently there are no formal national CVD prevention guidelines in Haiti. The Ministry of Health has prioritized hypertension screening, diagnosis and treatment as a national policy, and is working with clinics to formalize screening and treatment algorithms. Some Haitian physicians report using the Pooled Cohort Equations, also known as the Atherosclerotic Cardiovascular Disease (ASCVD) risk estimator, in clinical practice to estimate 10-year CVD risk [8]. However, available CVD risk prediction models are built on predominantly Caucasian, high-income country populations, and have not been evaluated in LMIC populations due to the paucity of rigorous prospective cohorts with adjudicated CVD outcomes [8–10]. Furthermore, model choice may have ramifications for which individuals are identified as high risk and recommended for treatment, with divergent cost and public health implications. There are limited population-based data describing statin eligibility for primary CVD prevention in Haiti.

The aim of this study is to compare the estimated 10-year risk of CVD across six commonly used CVD prediction models, and to identify high-risk groups for targeted statins. By applying these models to a population-based cohort in Haiti, we hope to identify if there is variation in the proportion of adults identified as high-risk which may reflect the need for models specific to populations living in low-income countries.

## Methods

### Study design

We used cross-sectional data within the Haiti CVD Cohort Study, a population-based cohort in Port-au-Prince selected using multistage random sampling with a previously described protocol (clinicaltrials.gov NCT03892265) [11]. This study has enrolled 3005 participants between March 2019 to August 2021 and follows them for 2–3.5 years to evaluate 1) the prevalence of traditional and poverty-related CVD risk factors, such as poor diet, smoking, hypertension, lead exposure, among others, and 2) the incidence of CVD events including myocardial infarction, angina, stroke, heart failure, and cardiac death. The six CVD risk prediction models in this analysis only estimate risk among adults ≥ 40 years because the underlying cohorts upon which the models are derived only include adults ≥ 40 years and because of the assumption that CVD risk factors likely do not become prevalent before 40 years of age [8–10]. Out of 3005 adults enrolled, 2890 (96.2%) had complete data required for the CVD risk prediction models. After excluding those < 40 years (*n* = 1397), already on a statin (*n* = 23), and with a history of myocardial infarction or stroke (*n* = 125), our final analytic sample was 1345 participants (Supplemental Fig. 1).

The study was conducted at the Groupe Haïtien d’Etude du Sarcome de Kaposi et des Infections Opportunistes clinics (GHESKIO), a medical organization that has operated continuously over four decades in Haiti to provide clinical care and conduct research on HIV and chronic diseases.

### Measurements

Demographic data (age, sex, education, income) and health behaviors (smoking status, physical activity) were collected during an enrollment survey using standardized WHO STEPwise Approach to NCD Risk Factor Surveillance instruments [12]. Clinical data, including height, weight, and blood pressure (BP), were measured during a physical exam with a study physician or nurse at enrollment.

BP was measured using the automated Omron HEM-907 machine with an appropriate cuff size (bladder encircling at least 80% of arm), after the participant had been seated in a quiet space for five minutes with both feet on the ground and their arm supported at heart level [12, 13]. Three BP measurements were taken on the left arm separated by one-minute intervals. In accordance with WHO guidelines, the second and third BP measurements were averaged for all analyses [12].

Medical history and diagnoses (hypertension, hyperlipidemia, diabetes, myocardial infarction, angina, stroke, HIV) were determined based on self-reported past medical history, direct imaging or laboratory measurement where applicable, and clinical evaluation performed by a trained study physician (Supplemental Table 1).

### CVD risk assessment and outcomes

Six models were compared: the Pooled Cohort Equations (PCEs) [8], an adjusted PCE (aPCE) incorporating updated cohorts with more African Americans [14], Framingham CVD Lipids [9], Framingham CVD Body Mass Index (BMI) [9], WHO-Lipids [10], and WHO-BMI [10] (Supplemental Table 2). These models were chosen because they are widely used, frequently compared in existing literature, and most include people of African descent. The systematic coronary risk evaluation (SCORE) model based on European cohorts was not used given it only predicts fatal CVD outcomes.

Underlying equations and coefficients were extracted from published literature and applied to the cohort [8–10, 14] (Supplemental Tables 3-5).

The primary outcome was predicted 10-year risk of CVD as 1) a continuous score and 2) a categorical score (low, intermediate, high). The secondary outcome was statin eligibility, based on model specific thresholds and criteria. For PCE and aPCE, statin eligibility for primary prevention included: 1) low density lipoprotein cholesterol (LDLc) ≥ 190 mg/dL, or 2) diabetes and LDLc ≥ 70 mg/dL, or 3) calculated 10-year CVD risk ≥ 7.5% and LDLc ≥ 70 mg/dL [15]. For Framingham equations, statin eligibility included: 1) LDLc ≥ 190 mg/dL, or 2) diabetes and LDLc ≥ 100 mg/dL, or 3) calculated 10-year CVD risk ≥ 20% and LDLc ≥ 100 mg/dL, or 4) 10-year CVD risk 10–20% and LDLc ≥ 130 mg/dL with ≥ 2 risk factors, or 5) 10-year CVD risk < 10% and LDLc ≥ 160 mg/dL with ≥ 2 risk factors [16]. Framingham risk factors for criteria 4 and 5 include: smoking, hypertension, high-density lipoprotein cholesterol (HDLc) < 40 mg/dL, myocardial infarction or angina in first degree relative before age 50, age ≥ 45 years for men, age ≥ 55 years for women. The WHO recommends tailoring statin eligibility within each country, but recommends statin eligibility for 10-year CVD risk ≥ 20% [7].

### Statistical analysis

For predicted 10-year risk of CVD, categorical scores were calculated using two methods: uniform thresholds (low < 5%, intermediate 5 to 7.5%, high ≥ 7.5%), and model specific thresholds (PCE and adjusted PCE: < 5%, 5 to 7.5%, ≥ 7.5%; Framingham-Lipids and Framingham-BMI: < 10%, 10 to 20%, ≥ 20%; WHO-Lipids and WHO-BMI: < 5%, 5 to 20%, ≥ 20%). Model specific thresholds exist due to differences in equation derivation, including measured CVD outcomes [8–10]. Spearman rank correlation coefficients were used to measure concordance between models’ ranked order of participants from lowest to highest risk, ranging from -1 (perfect discordance) to + 1 (perfect concordance). Categorical scores were compared using chi square tests of independence. Discordance was defined as participants categorized as low risk by one score, but high risk by another.

For participants categorized as high-risk using uniform thresholds, the underlying risk factors were summarized using medians, counts, and percentages to understand what risk factors were leading to the high-risk scores.

Statin eligibility was compared using chi square tests of independence. 95% confidence intervals (CI) were calculated using one sample proportions test.

All analyses were conducted using R, version 4.0.2.

## Results

Out of 3005 adults ≥ 18 years enrolled during the study period, 2890 (96.2%) had complete data and 1345 (44.8%) met study eligibility criteria. Of these 1345, 60.9% were female, 66.8% lived on a daily income of ≤ 1 USD, 52.9% had hypertension, 14.9% had hypercholesterolemia, 7.8% had diabetes mellitus, 4.0% were current smokers, and 2.5% had HIV (Table 1). Overall, 33.5% had a LDLc ≥ 130 mg/dL, 39.8% had a systolic blood pressure ≥ 140 mmHg, and 25.9% had a diastolic blood pressure ≥ 90 mmHg.

**Table 1:** Demographic and clinical characteristics of Haiti CVD Cohort (*N* = 1345)

	N (%), or median [IQR]
**Female**	819 (60.9)
**Age**, median [IQR], y	54 [47, 62]
**Education,** primary or lower	814 (60.5)
**Works for pay**	491 (36.5)
**Income (daily),** ≤ 1 USD	898 (66.8)
Comorbidities*	
Hypertension	712 (52.9)
On treatment	252 (18.7)
Hypercholesterolemia	200 (14.9)
On treatment	0
Diabetes Mellitus	105 (7.8)
On treatment	58 (4.3)
HIV	33 (2.5)
**Smoking,** current	54 (4.0)
**Physical Activity,** ≤ 150 min / week (low)	775 (57.7)
**Alcohol intake,** more than 1 drink a day (moderate-high)	30 (2.2)
**BMI ≥ 30 kg/m2**	293 (21.8)
**Cholesterol**	
HDL Cholesterol < 40 mg/dL	282 (21.0)
LDL Cholesterol ≥ 130 mg/dL	451 (33.5)
**Blood Pressure**	
SBP ≥ 140 mmHg	535 (39.8)
SBP ≥ 130 mmHg	736 (54.7)
DBP ≥ 90 mmHg	348 (25.9)
DBP ≥ 80 mmHg	644 (47.9)

### Predicted 10-year CVD risk

Using a continuous score, median predicted 10-year CVD risk ranged from 3.6% in the adjusted PCE model (IQR 1.7–8.2) to 9.6% in the Framingham-BMI model (IQR 4.9–18.0) (Table 2). Using the Spearman rank correlation coefficient, we assessed the concordance between how each model ranked each individual participant in order from lowest risk to highest risk in pairwise comparisons. Spearman coefficients showed high concordance between models, ranging from 0.86 (Framingham-Lipids vs WHO-BMI) to 0.98 (PCE vs adjusted PCE).

**Table 2:** Predicted 10-year CVD risk in Haiti CVD Cohort

	Haitian cohort (*N* = 1345)
CVD Risk Estimation Method	N (%)
**Pooled Cohort Equations (PCE)**	
Median (25th to 75th percentile)	6.1 [2.7, 12.2]
Low risk (< 5%)	588 (43.7)
Intermediate (5 to < 7.5%)	200 (14.9)
High (≥ 7.5%)	557 (41.4)
Statin eligibility^a^	563 (41.9)
**adjusted Pooled Cohort Equations (adjusted PCE)**	
Median (25th to 75th percentile)	3.6 [1.7, 8.2]
Low risk (< 5%)	813 (60.4)
Intermediate (5 to < 7.5%)	164 (12.2)
High (≥ 7.5%)	368 (27.4)
Statin eligibility^a^	408 (30.3)
**Framingham-Lipids**	
Median (25th to 75th percentile)	8.2 [4.2, 16.4]
Low (< 10%)	776 (57.7)
Intermediate (10 to < 20%)	327 (24.3)
High (≥ 20%)	242 (18.0)
Statin eligibility^a^	344 (25.6)
**Framingham-BMI**	
Median (25th to 75th percentile)	9.6 [4.9, 18.0]
Low (< 10%)	689 (51.2)
Intermediate (10 to < 20%)	360 (26.8)
High (≥ 20%)	296 (22.0)
Statin eligibility^a^	362 (27.0)
**WHO-Lipids**	
Median (25th to 75th percentile)	4.0 [2.0, 8.0]
Low risk (< 5%)	685 (50.9)
Intermediate-high-risk (5 to < 20%)	627 (46.6)
High (≥ 20%)	33 (2.5)
Statin eligibility^a^	33 (2.5)
**WHO-BMI**	
Median (25th to 75th percentile)	4.0 [2.0, 8.0]
Low risk (< 5%)	678 (50.4)
Intermediate-high-risk (5 to < 20%)	643 (47.8)
High (≥ 20%)	24 (1.8)
Statin eligibility^a^	24 (1.8)

*Legend:* ^a^ statin eligibility criteria by each model is detailed in the Supplement. 95% CI calculated using one sample proportions test.

**Fig. 1:**
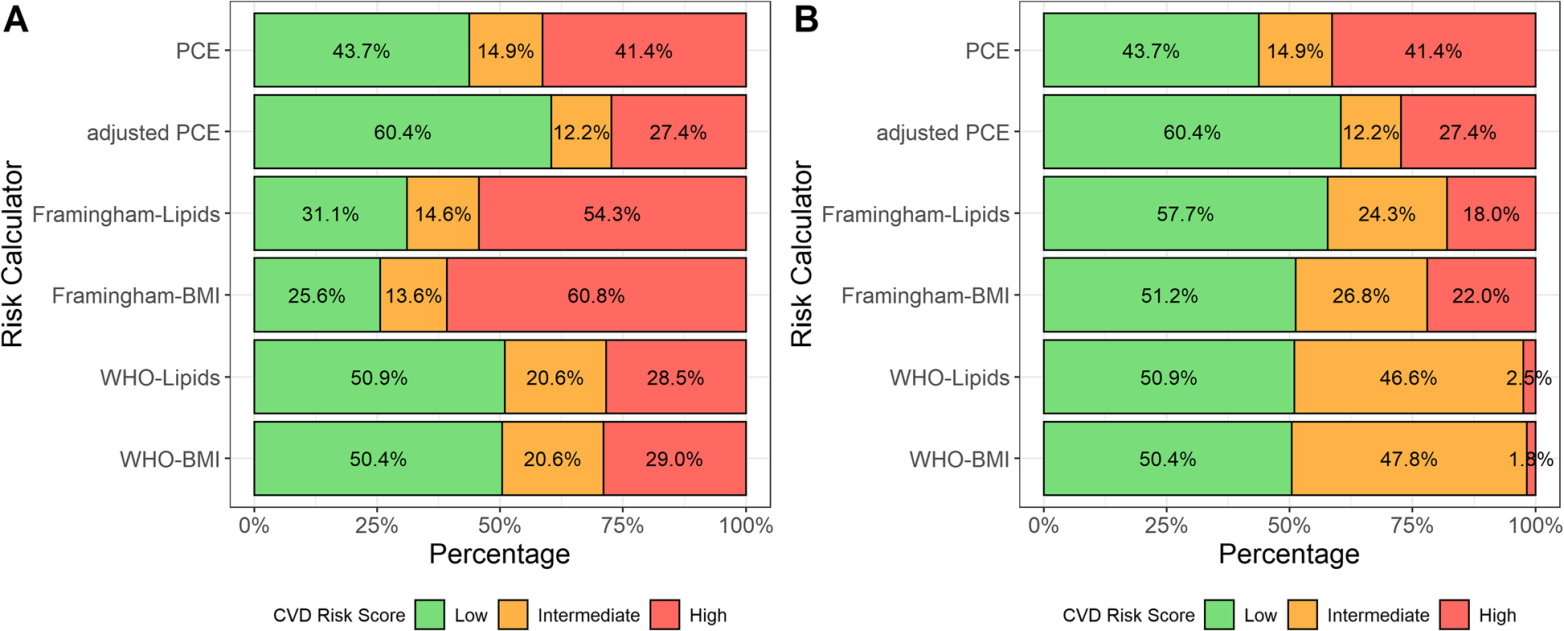
Predicted 10-year CVD risk categorizations by model. Legend: Figure shows proportion of cohort categorized as low, intermediate, or high-risk. Panel A uses a uniform threholds for low, intermediate, and high-risk: < 5%, 5 to 7.5%, and ≥ 7.5%. Panel B uses model specific thresholds for low, intermediate, and high-risk: PCE < 5%, 5 to 7.5%, ≥ 7.5%; adjusted PCE < 5%, 5 to 7.5%, ≥ 7.5%; Framingham-Lipids < 10%, 10 to 20%, ≥ 20%; Framingham-BMI < 10%, 10 to 20%, ≥ 20%; WHO-Lipids < 5%, 5 to 20%, ≥ 20%; WHO-BMI < 5%, 5 to 20%, ≥ 20%

However, categorization of individuals into risk groups using uniform thresholds showed extremely wide variability. The percent of the cohort categorized as high-risk ranged from 27.4% in the adjusted PCE model to 60.8% in the Framingham-BMI model (χ^2^ = 673, *p* value < 0.001) (Fig. 1A). Under uniform thresholds, 384 participants had discordant scores (categorized as high risk by one score, but low risk by another). The most common pattern was categorization as high risk by Framingham-lipids or Framingham-BMI and low risk by another model (380 out of 384 discordant participants).

Using model specific thresholds resulted in similarly wide variability in risk categorization (Table 2, Fig. 1B). The percent of the cohort categorized as high-risk ranged from 1.8% in WHO-BMI to 41.4% in PCE (χ^2^ = 1416, *p* value < 0.001). Under model specific thresholds, 122 participants had discordant scores, with the most common pattern as high risk by PCE and low risk by another model (118 out of 122 discordant participants).

### Risk factor distribution in high-risk category and statin eligibility

The risk factor distribution of age, sex, comorbidities, SBP, total cholesterol, and HDLc for participants with high 10-year CVD risk are summarized in Table 3. The median age ranged from 59 to 66, and percent female was 44.0% to 53.3%. Diabetes and current smoking were not common (< 20% and < 10%, respectively) in the high-risk groups. However, SBP was relatively high. Treated SBP, or participants taking antihypertensive medications, ranged from a median of 151 to 161 mmHg, and untreated SBP ranged from a median of 142 to 159 mmHg. Total cholesterol was also high, ranging from a median of 192 to 199 mg/dL.

**Table 3:** Risk factor distribution in high-risk category, using model-specific thresholds

	PCE	adjusted PCE	Framingham- Lipids	Framingham- BMI	WHO-Lipids	WHO-BMI
Percent of cohort categorized as high-risk by risk calculator	41.4%	27.4%	18.0%	22.0%	2.5%	1.8%
n	557	368	731	818	390	39
**Female,** n (%)	295 (53.0)	162 (44.0)	375 (51.3)	420 (51.3)	208 (53.3)	208 (53.3)
**Age**, median [25th to 75th percentile], y	62 [57, 68]	63 [57, 69]	60 [54, 66]	59 [53, 65]	66 [61, 70]	66 [61, 70]
Diabetes Mellitus**,** n (%)	80 (14.4)	68 (18.5)	94 (12.9)	96 (11.7)	35 (9.0)	35 (9.0)
Current smoker**,** n (%)	39 (7.0)	31 (8.4)	45 (6.2)	46 (5.6)	30 (7.7)	30 (7.7)
SBP treated, median [25th to 75th percentile], mmHg	155 [144, 172]	157 [146, 176]	152 [141, 169]	151 [138, 168]	161 [150, 179]	161 [150, 179]
SBP not treated, median [25th to 75th percentile], mmHg	146 [133, 162]	159 [144, 173]	143 [130, 158]	142 [129, 157]	148 [136, 166]	148 [136, 166]
**Total Cholesterol,** median [25th to 75th percentile], mg/dL	198 [170, 223]	198 [171, 224]	199 [171, 224]	192 [164, 218]	197 [168, 221]	197 [168, 221]
**HDL Cholesterol,** median [25th to 75th percentile], mg/dL	47 [40, 55]	47 [40, 55]	47 [40, 55]	48 [41, 56]	50 [43, 59]	50 [43, 59]

*Legend:* Model specific thresholds of high 10-year CVD risk were used to identify high-risk participants: PCE ≥ 7.5%; adjusted PCE ≥ 7.5%; Framingham-Lipids ≥ 20%; Framingham-BMI ≥ 20%; WHO-Lipids ≥ 20%; WHO-BMI ≥ 20%.

Using model specific thresholds, statin eligibility varied from 1.8% (95% CI 1.2% to 2.6%) with WHO-BMI to 41.4% (95% CI 39.2% to 44.5%) with PCE (χ^2^ = 1029, *p* value < 0.001) (Table 2).

## Discussion

Correctly identifying high-risk patients allows for targeted interventions for primary prevention of CVD and treatment of underlying risk factors. In the Haiti CVD Cohort, we found substantial variation in the proportion identified as high-risk using existing models, ranging from 1.8% to 41.4% using model-specific thresholds and 27.4% to 60.8% using a uniform threshold. Anywhere from 1.8% to 41.4% of participants were eligible for statins, with the PCE model resulting in the largest proportion eligible for statins, leading to very different treatment recommendations and public health implications depending on which prediction model is chosen.

Our study fills a critical gap in the literature—the lack of population-based studies to evaluate the variation of existing CVD risk prediction models in low-income countries, and Haiti specifically. This analysis is the first to report population-based estimates for high CVD risk and statin eligibility in Haiti using rigorous individual-level blood pressure lipid measurements. The WHO STEPs has not been conducted in Haiti, and the 2016 Haiti Demographic Health Survey does not include individual-level BP nor lipids data [17].

Our findings of substantial variation across CVD risk models are similar to other studies in LMICs, which include clinic and hospital-based cohorts or convenience samples. In an all-male Brazilian cohort, another country in the Caribbean-Latin America Region, 5.5% of men were high risk using the Framingham-Lipids model vs 0% using the PCE model [18]. A cohort in India comparing Framingham, PCE, and WHO found that 51.9%, 28.3%, and 16.2% were high risk, respectively [19]. In a cohort of HIV-infected patients in Botswana, Framingham classified 2.6% as high risk versus PCE at 14.1% [20]. Lastly, a study in Iran reported Framingham classified 8% men and 2% women as high risk, compared to 13% men and 6% women using PCE [21].

There are multiple potential reasons for the variation in proportion identified as high risk. First, these existing models do not include poverty and poverty-based risk factors that may be leading drivers of CVD in LMICs. Second, existing CVD risk models are built on largely Caucasian populations, and may not be accurate for a majority black LMIC population like Haiti or many sub-Saharan African countries. Traditional methods using Cox proportional hazards may overfit the data on small subgroups like African Americans, leading to inaccurate predictions, and require assumptions about proportional hazards which may not be true [14]. Newer statistical techniques, like machine learning, may avoid these limitations and integrate a larger breadth of data [22]. Prospective cohorts representative of LMIC with hard CVD outcomes are also needed to supply accurate underlying data. Lastly, different CVD risk models predict slightly different outcomes. However, the PCE, aPCE, WHO-Lipids and WHO-BMI predict essentially the same outcomes of CVD death, non-fatal MI, and non-fatal stroke [8, 10, 14]. Framingham-Lipids and Framingham-BMI have a more expanded list of predicted outcomes (Supplemental Table 2), but this is partially addressed by the use of a higher model-specific threshold for high risk (≥ 20%) [9].

There is an urgent need to design and validate CVD risk prediction tools in LMICs that include locally relevant risk factors reflecting relevant risk factors, cardiovascular disease pathology, and usability in low-resource settings. While ischemic heart disease accounts for the majority of CVD in high income countries (HIC), nonatherosclerotic stroke, hypertensive heart disease, and nonischemic cardiomyopathies are more common in LMIC [23]. In our cohort, examining the high-risk group across models showed hypertension was relatively common, while diabetes and smoking were not. Designing new CVD risk models will also require a focus on usability. Lipids are not routinely available in many places [24], making non-lab based methods such as those using BMI more feasible. While online CVD risk calculators are widely available in HIC, lack of reliable internet and friction of integration into busy workflows suggest paper-based wallcharts, such as produced by the WHO, may work better in LMIC.

To achieve desired health outcomes, CVD risk prediction must be translated into successful action, involving multisector action from health systems, health care providers, and patients. Our study is novel in describing how many people are statin eligible using existing CVD risk prediction models in a Haitian population-based cohort. Statin accessibility is low in many LMICs. Based on the WHO Health Action International survey, statins are not on the essential medicine list of 34% of countries, including Haiti [25, 26]. In Haiti, a 2011 survey showed atorvastatin and simvastatin were available in retail pharmacies, but rarely in public or nonprofit pharmacies, and expensive [27]. The lowest paid government worker would need 2.6 days wages to pay for a 1 month supply of statins if bought from a public sector pharmacy, and 13.7 day wages if bought from a retail pharmacy [27]. Lower availability and affordability of essential CVD meds have been associated with higher risk of major adverse cardiovascular events and mortality (HR 1.25, 95% CI 1.08 to 1.50) [28].

Strengths of this study include the use of a population-based cohort, research-grade BP measurement, and standardized lipid measurement. Limitations include the exclusion of young participants < 40 for whom traditional CVD risk models do not apply and yet may be at high risk in low-income countries where early-onset CVD risk factors have been reported, the cross-sectional design, and the lack of adjudicated CVD outcomes in prospective longitudinal data to compare predicted versus observed CVD events.

## Conclusions

In summary, across six commonly used CVD risk prediction models, there was substantial variation in identification of high-risk participants using both uniform, and model specific thresholds. By applying these models to a population-based cohort in Haiti, we hope to inform future prospective analyses with incident CVD data to determine which CVD risk factors should be used to optimize CVD risk prediction in a LMIC context. Locally relevant CVD risk prediction models are needed in LMIC, combined with health systems strengthening to increase treatment availability and affordability.

## Supplementary Information

**Additional file 1.** 

## Data Availability

Deidentified data used for this analysis are available upon request after signing a data access and use agreement, provision of approval by the GHESKIO ethics board, and demonstration that the external investigative team is qualified and has documented evidence of human research protection training. Researchers should provide a methodologically sound proposal. Requests may be addressed to mam9365@med.cornell.edu or irb@med.cornell.edu. Data are available following publications through 3 years after publication and will be provided directly from the PI.

## Abbreviations

aPCE: Adjusted pooled cohort equations
BMI: Body mass index
BP: Blood pressure
CVD: Cardiovascular disease
DBP: Diastolic blood pressure
GHESKIO: Groupe Haïtien d’Etude du Sarcome de Kaposi et des Infections Opportunistes clinics
IQR: Interquartile range
LMIC: Low middle income countries
PCE: Pooled cohort equations
SBP: Systolic blood pressure
SCORE: Systematic coronary risk evaluation model
WHO: World Health Organization
