# Supplementary material for: Comparing six cardiovascular risk prediction models in Haiti: implications for identifying high-risk individuals for primary prevention
Source: BMC Public Health. 2022 Mar 19;22:549. doi: 10.1186/s12889-022-12963-x (PMC8933947; doi:10.1186/s12889-022-12963-x)
Supplement: Supplementary file 1 — Additional file 1. [file 12889_2022_12963_MOESM1_ESM.docx]

**Supplemental Online Content for**: INSERT HERE

**Authors:** Lily D Yan MD MSc ^1,2^, Jean Lookens Pierre MD^3^, Vanessa Rouzier MD ^2, 3^, Michel Théard MD^4^, Alexandra Apollon MT^3^, Stephano St-Preux MS^3^, Justin R Kingery MD PhD^1,2^, Kenneth A Jamerson MD^5^, Marie Deschamps MD^3^, Jean W Pape MD^2,3^, Monika M Safford MD^1^, Margaret L McNairy MD MSc^1,2^

**Corresponding Author**

Lily D Yan, [liy9032@med.cornell.edu](mailto:liy9032@med.cornell.edu)

**Table of Contents:**

[Supplemental Figure 1 2](#_Toc88301536)

[Supplemental Table 1 3](#_Toc88301537)

[Methodology for CVD risk prediction application 4](#_Toc88301538)

[Supplemental Table 2: Summary of cardiovascular disease risk equations, populations, inputs, and outcomes 5](#_Toc88301539)

[Supplemental Table 3: Pooled Cohort Equation coefficients 6](#_Toc88301540)

[Supplemental Table 4: Adjusted Pooled Cohort Equation coefficients 7](#_Toc88301541)

[Supplemental Table 5: Framingham Lipids and Framingham BMI coefficients 8](#_Toc88301542)

[References 9](#_Toc88301543)

[STROBE Statement—Checklist of items that should be included in reports of *cross-sectional studies* 11](#_Toc88301544)

# Supplemental Figure 1


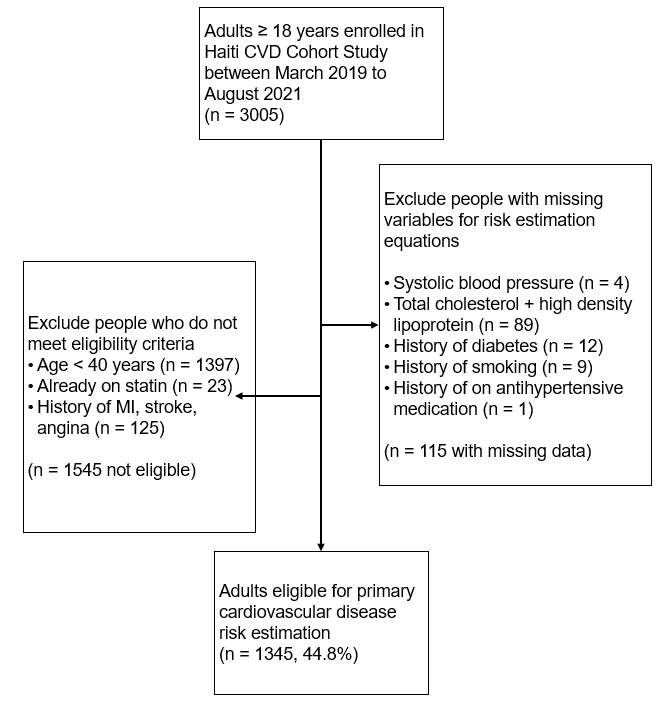


# Supplemental Table 1

| **Condition** | **Clinical or laboratory measurement** |
| --- | --- |
| Hypertension | - Study physician diagnosed patient with hypertension based on history and physical - Patient reports taking medication for high blood pressure - Average systolic blood pressure of last two out of three measurements ≥ 140 mmHg OR average diastolic blood pressure of last two out of three measurements ≥ 90 mmHg |
| Hypercholesterolemia | - Study physician diagnosed patient with hypercholesterolemia based on history and physical - Patient reports taking medication for hypercholesterolemia - non-HDLc 190-219 mg/L, OR LDLc ≥160 mg/dL ^1^. To convert LDLc to millimoles per liter, multiply by 0.0259 |
| Diabetes | - Study physician diagnosed patient with diabetes based on history and physical - Patient reports taking medication for diabetes - random glucose level ≥ 200 mg/dL OR fasting glucose level ≥ 126 mg/dL |

# Methodology for CVD risk prediction application

CVD risk prediction models are largely based on Cox proportional hazard models, developed using data from large population cohorts. The exception to this is the adjusted Pooled Cohort Equation (PCE) model developed by Yadlowsky et al ^2^, which uses newer statistical methods (elastic net regularization selection of logistic equations) to avoid the specific weaknesses of the original PCE derivation process: overfitting in small subpopulations like black adults, and violation of the proportional hazards assumption which affects the accuracy of risk estimates among subgroups.

The general process of calculating an individual’s 10 year CVD risk is as follows:

Step 1: Find underlying equation and coefficients from published literature ^1–5^

Step 2: Construct equations in R, building off previously published work ^2^

Step 3: Calculate CVD risk for each individual in cohort

Step 4: As a quality check, take a random 10% sample and manually calculate CVD risk using publicly available online calculators for PCE, adjusted PCE, Framingham Lipids, and Framingham BMI. Used publicly available wallcharts for WHO Lipids and WHO BMI. Compared manually calculated scores to values obtained in Step 3, and correct errors in equations as needed

The specific coefficients used from the published literature are detailed in Supplemental Tables 3-5. For the WHO Lipids and WHO BMI risk calculation, while the coefficients and equations are publicly available, Kaptoge et al ^5^ used Global Burden of Disease incident statistics to derive two rescaling factors for each world region, and then present a recalibrated 10 year CVD risk. However, these rescaling factors are not available publicly. Instead, we used the published WHO wallcharts to write a program in R (modeled off the whoishRisk package) that takes the inputs of sex, diabetes, smoking status, age, systolic blood pressure, and either lipids or BMI, and outputs the recalibrated risk based on the wallchart.^5^

## Supplemental Table 2: Summary of cardiovascular disease risk equations, populations, inputs, and outcomes

| Score | Population | Inputs | Outcome |
| --- | --- | --- | --- |
| Pooled Cohort  Equations  (ASCVD) ^1,3^ | U.S. population ages 40 to 79 drawn from Atherosclerosis Risk in Community Study (ARIC) ^6^, Cardiovascular Heart Study (CHS) ^7^, Coronary Artery Risk Development in Young Adults Study (CARDIA) ^8^, Framingham (1968 to 1987) ^9^, Framingham Offspring Study Cohorts ^10^ | Age, sex, race, diabetes, smoking, treatment for hypertension,  systolic blood pressure,  total cholesterol, high density lipoprotein cholesterol | Non-fatal MI, CHD death,  or fatal or non-fatal  stroke |
| Basu updated Pooled Cohort Equations ^2^ | U.S. population ages 40 to 79 drawn from Atherosclerosis Risk in Community Study (ARIC) ^6^, Cardiovascular Heart Study (CHS) ^7^, Coronary Artery Risk Development in Young Adults Study (CARDIA) ^8^, Framingham Offspring Study Cohorts ^10^, Jackson Heart Study (JHS) ^11^, Multi-Ethnic Study of Atherosclerosis (MESA) ^12^ | Age, sex, race, diabetes, smoking, treatment for hypertension,  systolic blood pressure,  total cholesterol, high density lipoprotein cholesterol | Non-fatal MI, CHD death, or fatal or non-fatal stroke |
| Framingham CVD lipids ^4^ | Framingham (1968 to 1987) ^9^, Framingham Offspring Study Cohorts ^10^ | Age, sex, smoking, diabetes,  systolic blood pressure, treatment for hypertension, total cholesterol, HLD cholesterol | CVD death, MI, coronary insufficiency, angina, ischemic stroke, hemorrhagic stroke, transient ischemic attack, peripheral artery disease, heart failure |
| Framingham CVD BMI ^4^ | Framingham (1968 to 1987) ^9^, Framingham Offspring Study Cohorts ^10^ | Age, sex, smoking, diabetes,  systolic blood pressure, treatment for hypertension, body mass index | CVD death, MI, coronary insufficiency, angina, ischemic stroke, hemorrhagic stroke, transient ischemic attack, peripheral artery disease, heart failure |
| WHO lipids ^5^ | Emerging Risk Factors Collaboration (ERFC) data with 85 cohorts ^13^, with model recalibration by Global Burden of Disease (GBD) study estimates and Non-Communicable Diseases Risk Factor Collaboration(NCD-RisC) estimates | Age, sex, smoking, diabetes, systolic blood pressure, total cholesterol | Fatal or non-fatal MI, CHD death, fatal or non-fatal stroke |
| WHO BMI ^5^ | Emerging Risk Factors Collaboration (ERFC) data with 85 cohorts ^13^, with model recalibration by Global Burden of Disease (GBD) study estimates and Non-Communicable Diseases Risk Factor Collaboration(NCD-RisC) estimates | Age, sex, smoking, diabetes, systolic blood pressure, body mass index | Fatal or non-fatal MI, CHD death, fatal or non-fatal stroke |

## Supplemental Table 3: Pooled Cohort Equation coefficients

|  | **Coefficients** | | | |  |
| --- | --- | --- | --- | --- | --- |
|  | Men | | Women | |  |
| **Independent variables** | White | African American | White | African American | |
| Ln Age | 12.344 | 2.469 | –29.799 | 17.114 | |
| Ln Age, squared | NA | NA | 4.884 | N/A | |
| Ln Total cholesterol (mg/dL) | 11.853 | 0.302 | 13.54 | 0.94 | |
| Ln Age × Ln Total Cholesterol | –2.664 | N/A | –3.114 | N/A | |
| Ln HDL-C (mg/dL) | –7.990 | –0.307 | –13.578 | –18.920 | |
| Ln Age × Ln HDL-C | 1.769 | N/A | 3.149 | 4.475 | |
| Ln Treated Systolic BP (mm Hg) | 1.797 | 1.916 | 2.019 | 29.291 | |
| Ln Age × Ln Treated Systolic BP | NA | NA | N/A | –6.432 | |
| Ln Untreated Systolic BP (mm Hg) | 1.764 | 1.809 | 1.957 | 27.82 | |
| Ln Age × Ln Untreated Systolic BP | NA | NA | N/A | –6.087 | |
| Current Smoker (1=Yes, 0=No) | 7.837 | 0.549 | 7.574 | 0.691 | |
| Ln Age × Current Smoker | –1.795 | N/A | –1.665 | N/A | |
| Diabetes (1=Yes, 0=No) | 0.658 | 0.645 | 0.661 | 0.874 | |
| Mean (Coefficient × Value) | 61.18 | 19.54 | –29.18 | 86.61 | |
| S_10_, or baseline survival estimate at 10 years | 0.9144 | 0.8954 | 0.9665 | 0.9533 | |

Legend: 10 year risk of CVD event= $1-{S_{10}}^{exp(\sum\beta_{x}-mean)}$, from Goff et al, 2013 ^3^.

## Supplemental Table 4: Adjusted Pooled Cohort Equation coefficients

|  | **Coefficients** | |
| --- | --- | --- |
| **Independent variables** | Men | Women |
| Age | 0.0642 | 0.106501 |
| Black race (1/0 for black/white) | 0.482835 | 0.43244 |
| Systolic blood pressure (mm Hg) squared | −0.000061 | 0.000056 |
| Systolic blood pressure | 0.03895 | 0.017666 |
| Taking blood pressure medication (1/0 for yes/no) | 2.055533 | 0.731678 |
| Diabetes mellitus (1/0 for yes/no) | 0.842209 | 0.94397 |
| Current smoker (1/0 for yes/no) | 0.895589 | 1.00979 |
| Ratio of total cholesterol (mg/dL) to high-density lipoprotein cholesterol (mg/dL) | 0.193307 | 0.151318 |
| Age if black (0 if not) | NA | −0.008580 |
| Systolic blood pressure if taking blood pressure medication (0 if not) | −0.014207 | −0.003647 |
| Systolic blood pressure if black (0 if not) | 0.011609 | 0.006208 |
| Black race and taking blood pressure medication (1/0 for yes/no) | −0.119460 | 0.152968 |
| Age × systolic blood pressure | 0.000025 | −0.000153 |
| Black race and diabetes mellitus (1/0 for yes/no) | −0.077214 | 0.115232 |
| Black race and current smoker (1/0 for yes/no) | −0.226771 | –0.092231 |
| Ratio of total cholesterol to high-density lipoprotein cholesterol if black | −0.117749 | 0.070498 |
| Systolic blood pressure if black and taking blood pressure medication (0 if not) | 0.00419 | −0.000173 |
| Age × systolic blood pressure if black (0 if not) | −0.000199 | −0.000094 |
| Mean (intercept) | −11.679980 | −12.823110 |

Legend: 10 year risk of CVD event=$\frac{1}{1+exp\left( -sum of terms \right)}$, from Yadlowsky et al, 2018 ^2^.

## Supplemental Table 5: Framingham Lipids and Framingham BMI coefficients

|  | **Coefficients** | |
| --- | --- | --- |
| **Independent variables** | Men | Women |
| **Framingham Lipids** |  |  |
| Log of Age | 3.06117 | 2.32888 |
| Log of Total Cholesterol | 1.1237 | 1.20904 |
| Log of HDL Cholesterol | -0.93263 | -0.70833 |
| Log of SBP if not treated | 1.93303 | 2.76157 |
| Log of SBP if treated | 1.99881 | 2.82263 |
| Smoking | 0.65451 | 0.52873 |
| Diabetes | 0.57367 | 0.69154 |
| Mean (Coefficient × Value) | 23.9802 | 26.1931 |
| S_10_, or baseline survival estimate at 10 years | 0.88936 | 0.95012 |
| **Framingham BMI** |  |  |
| Log of Age | 3.11296 | 2.72107 |
| Log of Body Mass Index | 0.79277 | 0.51125 |
| Log of SBP if not treated | 1.85508 | 2.81291 |
| Log of SBP if treated | 1.92672 | 2.88267 |
| Smoking | 0.70953 | 0.61868 |
| Diabetes | 0.5316 | 0.77763 |
| Mean (Coefficient × Value) | 23.9388 | 26.0145 |
| S_10_, or baseline survival estimate at 10 years | 0.88431 | 0.94833 |

Legend: 10 year risk of CVD event = $1-{S_{10}}^{exp(\sum\beta_{x}-mean)}$, from D’Agostino et al, 2008 ^4^.

# References

1. Grundy SM, Stone NJ, Bailey AL, et al. 2018 AHA/ACC/AACVPR/AAPA/ABC/ACPM/ADA/AGS/APhA/ASPC/NLA/PCNA Guideline on the Management of Blood Cholesterol: A Report of the American College of Cardiology/American Heart Association Task Force on Clinical Practice Guidelines. *Circulation*. 2019;139(25):e1082-e1143. doi:10.1161/CIR.0000000000000625

2. Yadlowsky S, Hayward RA, Sussman JB, McClelland RL, Min YI, Basu S. Clinical Implications of Revised Pooled Cohort Equations for Estimating Atherosclerotic Cardiovascular Disease Risk. *Ann Intern Med*. 2018;169(1):20. doi:10.7326/M17-3011

3. Goff DC, Lloyd-Jones DM, Bennett G, et al. 2013 ACC/AHA guideline on the assessment of cardiovascular risk: a report of the American College of Cardiology/American Heart Association Task Force on Practice Guidelines. *Circulation*. 2014;129(25 Suppl 2):S49-73. doi:10.1161/01.cir.0000437741.48606.98

4. D’Agostino RB, Ramachandran VS, Pencina MJ, et al. General Cardiovascular Risk Profile for Use in Primary Care. *Circulation*. 2008;117(6):743-753. doi:10.1161/CIRCULATIONAHA.107.699579

5. Kaptoge S, Pennells L, Bacquer DD, et al. World Health Organization cardiovascular disease risk charts: revised models to estimate risk in 21 global regions. *Lancet Glob Health*. 2019;7(10):e1332-e1345. doi:10.1016/S2214-109X(19)30318-3

6. THE ARIC INVESTIGATORS. THE ATHEROSCLEROSIS RISK IN COMMUNIT (ARIC) STUI)Y: DESIGN AND OBJECTWES. *Am J Epidemiol*. 1989;129(4):687-702. doi:10.1093/oxfordjournals.aje.a115184

7. Fried LP, Borhani NO, Enright P, et al. The cardiovascular health study: Design and rationale. *Ann Epidemiol*. 1991;1(3):263-276. doi:10.1016/1047-2797(91)90005-W

8. Friedman GD, Cutter GR, Donahue RP, et al. Cardia: study design, recruitment, and some characteristics of the examined subjects. *J Clin Epidemiol*. 1988;41(11):1105-1116. doi:10.1016/0895-4356(88)90080-7

9. Dawber TR, Kannel WB, Lyell LP. An approach to longitudinal studies in a community: the Framingham Study. *Ann N Y Acad Sci*. 1963;107:539-556. doi:10.1111/j.1749-6632.1963.tb13299.x

10. KANNEL WB, FEINLEIB M, McNAMARA PM, GARRISON RJ, CASTELLI WP. AN INVESTIGATION OF CORONARY HEART DISEASE IN FAMILIES: THE FRAMINGHAM OFFSPRING STUDY. *Am J Epidemiol*. 1979;110(3):281-290. doi:10.1093/oxfordjournals.aje.a112813

11. Sempos CT, Bild DE, Manolio TA. Overview of the Jackson Heart Study: a study of cardiovascular diseases in African American men and women. *Am J Med Sci*. 1999;317(3):142-146. doi:10.1097/00000441-199903000-00002

12. Bild DE, Bluemke DA, Burke GL, et al. Multi-Ethnic Study of Atherosclerosis: objectives and design. *Am J Epidemiol*. 2002;156(9):871-881. doi:10.1093/aje/kwf113

13. Emerging Risk Factors Collaboration, Danesh J, Erqou S, et al. The Emerging Risk Factors Collaboration: analysis of individual data on lipid, inflammatory and other markers in over 1.1 million participants in 104 prospective studies of cardiovascular diseases. *Eur J Epidemiol*. 2007;22(12):839-869. doi:10.1007/s10654-007-9165-7

**STROBE Checklist**

# STROBE Statement—Checklist of items that should be included in reports of *cross-sectional studies*

|  | Item No | Recommendation | Page No |
| --- | --- | --- | --- |
| **Title and abstract** | 1 | (*a*) Indicate the study’s design with a commonly used term in the title or the abstract | 1 |
|  |  | (*b*) Provide in the abstract an informative and balanced summary of what was done and what was found | 2-3 |
| Introduction | | | |
| Background/rationale | 2 | Explain the scientific background and rationale for the investigation being reported | 4 |
| Objectives | 3 | State specific objectives, including any prespecified hypotheses | 4 |
| Methods | | | |
| Study design | 4 | Present key elements of study design early in the paper | 4-5 |
| Setting | 5 | Describe the setting, locations, and relevant dates, including periods of recruitment, exposure, follow-up, and data collection | 4 |
| Participants | 6 | (*a*) Give the eligibility criteria, and the sources and methods of selection of participants | 4-6 |
| Variables | 7 | Clearly define all outcomes, exposures, predictors, potential confounders, and effect modifiers. Give diagnostic criteria, if applicable | 4-6 |
| Data sources/ measurement | 8* | For each variable of interest, give sources of data and details of methods of assessment (measurement). Describe comparability of assessment methods if there is more than one group | 4-6 |
| Bias | 9 | Describe any efforts to address potential sources of bias | 4-6 |
| Study size | 10 | Explain how the study size was arrived at | 4-5 |
| Quantitative variables | 11 | Explain how quantitative variables were handled in the analyses. If applicable, describe which groupings were chosen and why | 4-6 |
| Statistical methods | 12 | (*a*) Describe all statistical methods, including those used to control for confounding | 6 |
|  |  | (*b*) Describe any methods used to examine subgroups and interactions | N/A |
|  |  | (*c*) Explain how missing data were addressed | 5-6 |
|  |  | (*d*) If applicable, describe analytical methods taking account of sampling strategy | N/A |
|  |  | (*e*) Describe any sensitivity analyses | 5-6 |
| Results | | | |
| Participants | 13* | (a) Report numbers of individuals at each stage of study—eg numbers potentially eligible, examined for eligibility, confirmed eligible, included in the study, completing follow-up, and analysed | 7 |
|  |  | (b) Give reasons for non-participation at each stage | 5-7 |
|  |  | (c) Consider use of a flow diagram | SFig 1 |
| Descriptive data | 14* | (a) Give characteristics of study participants (eg demographic, clinical, social) and information on exposures and potential confounders | 7, Table 1 |
|  |  | (b) Indicate number of participants with missing data for each variable of interest | 7, Table 1 |
| Outcome data | 15* | Report numbers of outcome events or summary measures | 7-8, Fig 1 |
| Main results | 16 | (*a*) Give unadjusted estimates and, if applicable, confounder-adjusted estimates and their precision (eg, 95% confidence interval). Make clear which confounders were adjusted for and why they were included | 7-8 |
|  |  | (*b*) Report category boundaries when continuous variables were categorized | 7-8 |
|  |  | (*c*) If relevant, consider translating estimates of relative risk into absolute risk for a meaningful time period | N/A |
| Other analyses | 17 | Report other analyses done—eg analyses of subgroups and interactions, and sensitivity analyses | 7-8 |
| Discussion | | | |
| Key results | 18 | Summarise key results with reference to study objectives | 9 |
| Limitations | 19 | Discuss limitations of the study, taking into account sources of potential bias or imprecision. Discuss both direction and magnitude of any potential bias | 10 |
| Interpretation | 20 | Give a cautious overall interpretation of results considering objectives, limitations, multiplicity of analyses, results from similar studies, and other relevant evidence | 9-10 |
| Generalisability | 21 | Discuss the generalisability (external validity) of the study results | 9-10 |
| Other information | | | |
| Funding | 22 | Give the source of funding and the role of the funders for the present study and, if applicable, for the original study on which the present article is based | online submission |
